# Altered molecular specificity of surfactant phosphatidycholine synthesis in patients with acute respiratory distress syndrome

**DOI:** 10.1186/s12931-014-0128-8

**Published:** 2014-11-07

**Authors:** Ahilanandan Dushianthan, Victoria Goss, Rebecca Cusack, Michael PW Grocott, Anthony D Postle

**Affiliations:** NIHR Respiratory Biomedical Research Unit, University Hospital Southampton NHS Foundation Trust, Southampton, SO16 6YD UK; Integrative Physiology and Critical Illness Group, Clinical and Experimental Sciences, Sir Henry Wellcome Laboratories, Faculty of Medicine, University of Southampton, Southampton, SO16 6YD UK; Critical Care Research & Anaesthesia Unit, CE 93, MP24, E-Level, Centre Block, University Hospital Southampton NHS Foundation Trust, Southampton, SO16 6YD UK; Department of Critical Care Unit, Portsmouth Hospitals NHS Trust, Queen Alexandra Hospital, Southwick Hill Road, Cosham, PO6 3LY UK

**Keywords:** Acute respiratory distress syndrome, Acute lung injury, Surfactant, Phosphatidylcholine, Deuterated Choline, Stable isotopes, Isotope labelling, Mass spectrometry

## Abstract

**Background:**

Acute respiratory distress syndrome (ARDS) is a life-threatening critical illness, characterised by qualitative and quantitative surfactant compositional changes associated with premature airway collapse, gas-exchange abnormalities and acute hypoxic respiratory failure. The underlying mechanisms for this dysregulation in surfactant metabolisms are not fully explored. Lack of therapeutic benefits from clinical trials, highlight the importance of detailed in-vivo analysis and characterisation of ARDS patients according to patterns of surfactant synthesis and metabolism.

**Methods:**

Ten patients with moderate to severe ARDS were recruited. Most (90%) suffered from pneumonia. They had an infusion of *methyl*-D_9_-choline chloride and small volume bronchoalveolar lavage fluid (BALF) was obtained at 0,6,12,24,48,72 and 96 hours. Controls were healthy volunteers, who had BALF at 24 and 48 hours after *methyl*-D_9_-choline infusion. Compositional analysis and enrichment patterns of stable isotope labelling of surfactant phosphatidylcholine (PC) was determined by electrospray ionisation mass spectrometry.

**Results:**

BALF of patients with ARDS consisted of diminished total PC and fractional PC16:0/16:0 concentrations compared to healthy controls. Compositional analysis revealed, reductions in fractional compositions of saturated PC species with elevated levels of longer acyl chain unsaturated PC species. Molecular specificity of newly synthesised PC fraction showed time course variation, with lower PC16:0/16:0 composition at earlier time points, but achieved near equilibrium with endogenous composition at 48 hours after *methyl*-D_9_-choline infusion. The enrichment of *methyl*-D_9_-choline into surfactant total PC is nearly doubled in patients, with considerable variation between individuals.

**Conclusions:**

This study demonstrate significant alterations in composition and kinetics of surfactant PC extracted from ARDS patients. This novel approach may facilitate biochemical phenotyping of ARDS patients according to surfactant synthesis and metabolism, enabling individualised treatment approaches for the management of ARDS patients in the future.

## Introduction

Surfactant complex is a mixture of phospholipids and proteins. Phosphatidylcholine is the major surfactant phospholipid and among the several species, PC16:0/16:0 is the principle PC, thought to be actively involved in surface reduction at the air-liquid interface [1]. In patients with respiratory failure secondary to acute respiratory distress syndrome (ARDS), the lavaged surfactant complex show compositional derangement and lacks adequate surface activity [2,3]. Consequently, these changes are likely to contribute to the detrimental clinical features of severe hypoxemia, poor lung compliance and lung atelectasis, which are characteristic of ARDS. However, the exact underlying mechanisms for this dysregulation in surfactant metabolism is not known. The plausible hypothesises are complex and includes decreased synthesis/ secretion, increased breakdown by hydrolysis or oxidation, increased clearance and or functional inhibition by invasion of non-surfactant material into the alveolus [4]. Human in-vivo studies exploring these mechanisms are lacking and consequently, there remains a significant gap in the understanding of surfactant metabolism during acute alveolar injury.

ARDS is a heterogeneous disease process with significant variations in aetiology, pathophysiology, response to therapy and outcome. Furthermore, it imposes significant morbidity and mortality and lack of defined pharmacotherapies to moderate disease process remains an enduring challenge. Current diagnostic definitions fail to identify a homogenous population, and are limited by the lack of specificity and information regarding possible underlying genophenotypes. The disappointing results from surfactant replacement clinical trials in adults, reiterate the necessity for in-vivo human studies to explore the exact underlying mechanisms leading to the disordered surfactant metabolism evident in ARDS. Such studies may enable an individualised treatment strategy in the future after biochemical phenotyping of patients according to their intrinsic surfactant metabolism.

Traditional assessments of pulmonary surfactant consisted of isolation with sequential bronchoalveolar lavage fluid (BALF) and subsequent separation of surfactant phospholipids by liquid chromatography [3,5]. Such studies provided significant insights into surfactant compositional abnormalities in patients with ARDS. However, quantitative phospholipid assessments were unpredictable due to variability in surfactant recovery by BALF. Furthermore, such approaches lack specific information regarding the dynamics of surfactant synthesis and turnover. Stable isotope labelling coupled with analytical methods of electrospray ionization mass spectrometry (ESI-MS/MS), provides a novel approach to the dynamic assessment of surfactant metabolism in addition to highly specific molecular analysis of surfactant phospholipids [6]. Consequently, we used this methodology to characterise surfactant phosphatidylcholine kinetics in patients with ARDS.

## Methods

### Study population

Following approval from National Research Ethics Committee (10/WNo01/52) and University Hospital Southampton Research and Development Department (CRI0244), patients with ARDS were identified and recruited from general intensive care unit at University Hospital Southampton NHS Foundation Trust. The diagnosis of ARDS was based on the American European Consensus Conference diagnostic criteria [7]. All eligible patients were enrolled after informed assent was obtained from patient’s next of kin within 72 hours of onset of ARDS. All patients were sedated and required invasive mechanical ventilation. The control group consisted of nine healthy volunteers without any prior history of lung diseases and were all non-smokers.

### *Methyl*-D_9_ choline chloride

Deuterated *methyl*-D_9_ choline chloride is a stable isotope of choline, which can be used to probe dynamic PC synthetic pathways in-vivo and has been successfully applied to quantify surfactant PC flux via the CDP-choline pathway in healthy human volunteers and in animal models [8,9]. Following informed assent or consent, patients and controls had an intravenous infusion of *methyl*-D_9_ choline chloride (3.6 mg/kg) for a three hour period.

### Sample collection and processing

Bronchoalveolar lavage fluid (BALF) was obtained via a fibre-optic bronchoscope (Olympus BF P60). All patients were intubated with an endotracheal tube (size 7–9) and mechanically ventilated. Prior to the bronchoscopy, patients were pre-oxygenated with 100% oxygen and this was continued throughout the procedure. Patients were given additional sedation if required to facilitate the procedure. Bronchoscopy was not performed on patients with inspired oxygen (Fi0_2_) of >80% and was abandoned, when there was a significant desaturation defined as pulse oximetry oxygen saturation of <85%. Small volumes of BALF were collected by suctioning after instillation of 10-20 mls of warm sterile saline from a single lobar bronchus (either from middle or lower lobes). This was then rotated (in the order of right middle lobe, left middle lobe, right lower lobe and left lower lobe) for the subsequent lavages to minimise theoretical concerns, that repeated lavage of the same lobe could be detrimental to surfactant concentration or function in that lobe. This small volume BALF was performed at baseline before the administration of *methyl*-D_9_-choline chloride infusion, and at 6, 12, 24, 48, 72, 96 hours after the infusion. The reason for the frequent sampling recovery in the early stages was to ensure adequate analysis is performed at this stage, which could be utilised to target therapeutic interventions. For healthy volunteers, small volume BALF was obtained at 24 and 48 hours after *methyl*-D_9_-choline infusion.

BALF was transferred at 4°C to the processing laboratory. 10ul of Butylated Hydroxy Toluene (BHT) (20 g/L) solution was added to all samples as an anti-oxidant. Then the samples were filtered through a 100um nylon cell strainer and centrifuged at 100 x g for 20 seconds at 4°C. The resultant liquid material was further centrifuged at 400 g for 10 minutes at 4°C. The supernatant was aliquoted in eppendorfs and stored in a −80°C freezer.

### Phospholipid extraction

Total lipid extraction was performed using the Bligh and Dyer method [10]. Samples were made up to a volume of 800ul with 0.9% NaCl and dimyristol-PC (PC14:0/14:0) was added as internal standard (IS). One ml of chloroform and 2 mls of methanol were added to the samples, followed by further 1 ml of chloroform and 1 ml of distilled water. Sharper resolution was achieved by centrifuging at 400 g at 20°C for 20 minutes. The lower lipid rich layer was then removed and dried under a nitrogen concentrator at 40°C. Once dried further 1 ml of chloroform was added and dried again to be analysed by mass spectrometry.

### Mass spectrometry analysis and data extraction

The dried phospholipid fraction was suspended in a mixture of methanol-butanol-water-concentrated NH_4_OH (6:2:1.6:0.4 v/v), and was injected by syringe pump at a rate of 8ul/ml into the electrospray ionisation interface of a Xevo TQ mass spectrometer (Waters Corporation, UK) (ESI-MS/MS). The endogenous and newly produced PC species were calculated from MS/MS fragmentations of precursor ion m/z +184 and m/z + 193 respectively [8,9]. Dedicated excel spread sheets were used to quantify ion peaks after corrections for ^13^C-isotopic effects.

### Statistical analysis

Data were summarised by means, standard deviation (SD) and standard errors of means (SEM). The difference in composition in each group was examined by calculating the difference in means. Single comparisons were analysed by Student’s t-test and multiple comparisons by ANOVA of variance.

## Results

### Patient characteristics

Ten patients with ARDS, including 5 males and 5 females with a mean age of 61 and a range of 38–90 were recruited. The mean Pa0_2_/Fi0_2_ ratio was 113 mmHg, suggesting moderate to severe hypoxic respiratory failure. The APACHE II score, which defines the overall severity of their illness, was 23.2. All patients were intubated for respiratory failure and were managed with invasive ventilation. The duration of mechanical ventilation was 8.9 days. The mean length of ICU and hospital stay was 12.4 and 18.6 days respectively. The ICU mortality was 50%.

The majority of patients presented with pneumonia (90%) and most (80%) needed at least one other organ support in addition to invasive mechanical ventilation. One patient did not survive beyond 24 hours from enrolment and hence, only had BALF samples up to 12 hours after *methyl*-D_9_-choline infusion. While all other patients had small volume BALF at least until 48 hours, some did not have theirs at later time points (72 and 96 hours) due to either planned extubation or death. All patients were enrolled within 72 hours of diagnosis of ARDS and the actual mean enrolment time was <48 hours. The summary of patient’s characteristics is listed in Table 1.

**Table 1:** **Baseline characteristics and outcomes of recruited ARDS patients**

**Patient’s characteristics**	
Age	61 (38–90)
M:F	5:5
Pa0_2_/Fi0_2_ ratio (mmHg)	113.4 (63.5–169.3)
Lung Injury Score	3.2 (2.5–3.5)
APACHE II Score	23.2 (17–30)
Tidal volumes (ml/kg IBW)	6.7 ± 1.3
PEEP (cm of H_2_0)	10.8 ± 3.1
Ventilation days	8.9 (3–19)
Length of ICU stay	12.4 (3–35)
Length of hospital stay	18.6 (9–53)
ICU survival	50%

M, male; F, female; Pa0_2_/Fi0_2_ ratio, ratio of partial pressure of arterial oxygen and fraction of inspired oxygen; APACHE, acute physiology and chronic health evaluation; IBW, ideal body weight; ICU, intensive care unit. Data expressed as mean ± standard deviation or mean (range).

Small volume BALF was tolerated by all without any significant immediate complications including pneumothoraxes or cardiac arrhythmias. Patients needed additional sedation with boluses of propofol (40 ± 3 mgs) or midazolam (2 ± 0.3 mgs) for this procedure. There were no significant step-up in oxygen requirement post procedure (Fi0_2_-pre 54 ± 10%, Fi0_2_-post 55 ± 9%). The oxygen saturation pre- procedure was 94 ± 2% and post procedure was 91 ± 3%.

### Mass spectrometry analysis of BALF PC

**Figure 1:**
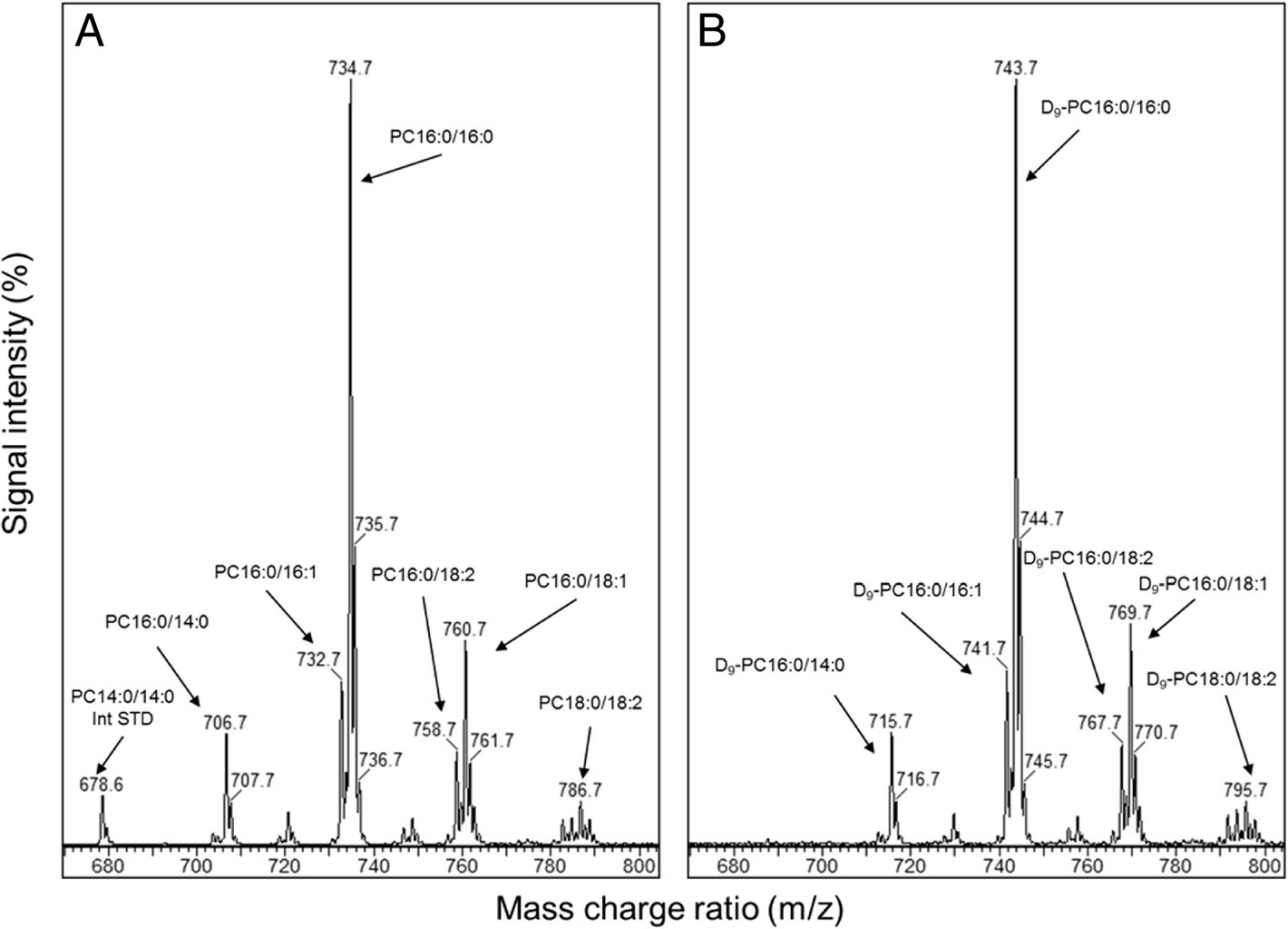
**Bronchoalveolar lavage phosphatidylcholine mass spectra from a healthy control for precursor scans of P184 (A) (endogenous phosphatidylcholine composition) and precursor scans of P193 (B) (newly synthesised deuterated phosphatidylcholine composition at 24 hours after** ***methyl*** **-D**_**9**_ **choline infusion).** The relative signal intensity of spectra B is nearly one hundredth of spectra A. The m/z 678 represents an internal standard of PC14:0/14:0. The *methyl*-D_9_-labelled phosphatidylcholine composition consisted of phosphatidylcholine species with 9 mass units higher than that of endogenous composition. PC, phosphatidylcholine; m/z, mass to charge ratio.

The molecular species of glycerophospholipids such as phosphatidylcholines can be classified according to the nature of esterified bond at the sn-1 (carbon-1) and sn-2 (carbon-2) positions of glycerol back bone, the number of carbon atoms in the fatty acid chains and the number of double bonds. The number of carbon atoms in the fatty acid chain determines the length of the chain and the number of double bonds in the side chain denotes their degree of saturation/unsaturation. For instance, dipalmitoyl PC is denoted as PC16:0/16:0, where there are two saturated fatty acids chains with sixteen carbons length. The BALF endogenous PC molecular composition was analysed by ESI-MS/MS from precursor scans of m/z 184 (P184). Subsequent enrichment of *methyl*-D_9_ choline, resulted in the generation of PC species with 9 mass units higher than those of endogenous composition. These were readily identified by precursor scans of m/z 193 (P193). A typical mass spectra for BALF PC endogenous and newly generated PC composition from controls is shown by Figure 1, with PC species for that particular mass charge ratio. In general, the most abundant PC species were PC16:0/16:0, PC16:0/18:1, PC16:0/14:0 and PC16:0/16:1. This distinctive PC composition mainly consisting of saturated PC species is a typical feature of pulmonary surfactant [1].

### Total Surfactant PC and PC16:0/16:0 concentration

**Figure 2:**
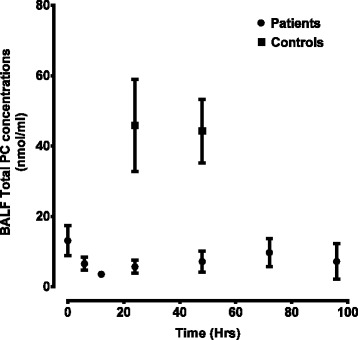
**Bronchoalveolar lavage total phosphatidylcholine concentrations over time for patients and controls.** Measured by sum of all phosphatidylcholine species present >1% abundance. [Controls (n) =9 for both time points, patients (n) =10, n = 9, n = 10, n = 9, n = 9, n = 8, n = 7 for time points 0,6,12,24,48,72 and 96 respectively]. Data expressed as mean ± SEM. BALF, bronchoalveolar lavage fluid; PC, phosphatidylcholine.

Total surfactant PC concentration was estimated by the sum of all PC species present at greater than 1% abundance corrected for recovery of PC14:0/14:0 internal standard. There was a significant reduction (P < 0.0001) in the total BALF PC isolated in patients compared to healthy controls at all-time points studied (Figure 2). At enrolment (T = 0 Hrs), the mean total PC concentration in the patient group was 13 ± 4 nmol/ml and accounted for only 30% of the controls. A further reduction in PC concentration was noted at 12 hours after enrolment. Additionally in patients, there was no significant recovery in total PC concentration over the investigative period up to 96 hours (Figure 2). PC16:0/16:0 concentrations were also much lower in patients at enrolment (2.6 ± 0.3 nmol/ml) and only accounted for about 10% of the healthy control values.

### Surfactant PC molecular composition at enrolment

**Figure 3:**
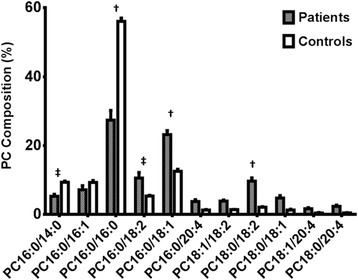
**Comparison of bronchoalveolar lavage surfactant phosphatidylcholine composition between patients at enrolment (T = 0 Hrs) and controls at earliest time point after enrolment (T = 24 Hrs).** Controls (n) =9 and patients (n) =10. Presented as mean ± SEM, †, P < 0.0001; ‡, P < 0.05. PC, phosphatidylcholine.

**Figure 4:**
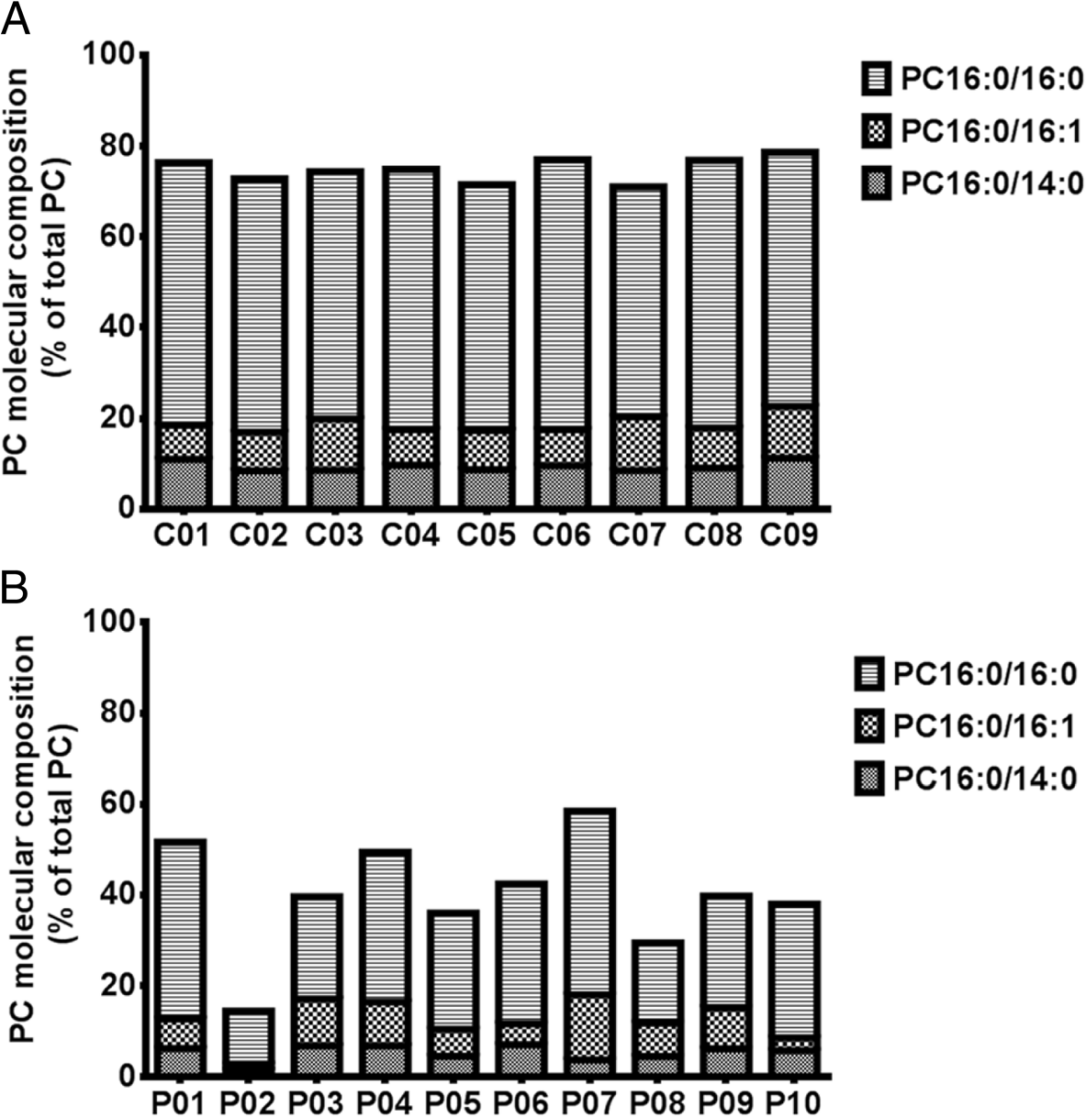
**Individual variations of surfactant specific phosphatidylcholine species (PC16:0/14:0), PC16:0/16:1 and PC16:0/16:0) between controls at earliest time point after enrolment (T=24 Hrs) (A), and patients at enrolment (T=0) (B).** Controls (n) =09 and patients (n) =10. PC, phosphatidylcholine; C, individual controls; P, individual patients.

At enrolment (T = 0 Hrs), surfactant PC composition of patients consisted of PC16:0/16:0 (27.4 ± 2.8%), PC16:0/18:1 (23.2 ± 1.2%), PC16:0/18:2 (10.6 ± 1.6%) and PC18:0/18:2 (9.7 ± 1.0%) with other PC species in much lesser abundance. Compared to the healthy controls, surfactant recovered from ARDS patients had significantly lower proportions of surfactant specific PC species, PC16:0/16:0 [mean difference (MD) of 29%, P < 0.0001] and PC16:0/14:0 (MD of 4% P = 0.02). The relative contribution of PC16:0/16:1 did not change between groups, while the longer acyl chain unsaturated PC species were elevated in patients [PC16:0/18:1 (MD 11%, P < 0.0001), PC16:0/18:2 (MD 5.2%, P = 0.0014) and PC18:0/18:2 (MD of 7.5% P < 0.0001, Figure 3). The saturated PC species characteristic of surfactant (PC16:0/16:0 and PC16:0/14:0) gradually increased for patients over time, with decreased relative concentration of unsaturated PC species (PC16:0/18:1 and PC18:0/18:2). However, despite these apparent improvements to surfactant composition, the fractional content of unsaturated PC species remained significantly elevated at 96 hours, with PC16:0/16:0 being still low at 40.6 ± 7.0%. The sum of surfactant specific PC species (PC16:0/16:0, PC16:0/14:0 and PC16:0/16:1) was consistently between 70-75% for healthy controls, but was widely variable for patients at enrolment (14- 59%, Figure 4).

### Total surfactant PC and fractional PC16:0/16:0 *methyl*- D_9_-choline enrichment

**Figure 5:**
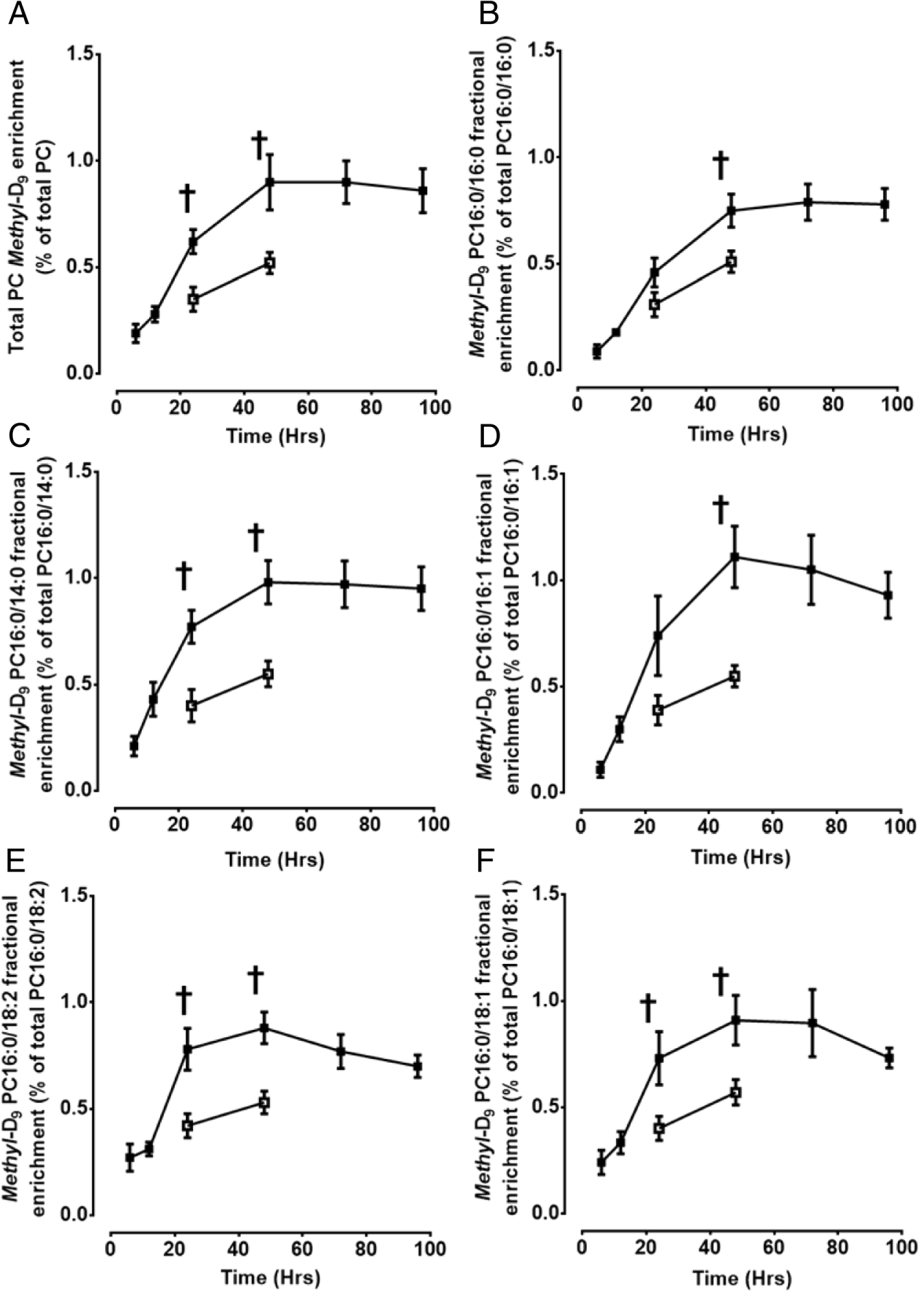
**Comparison of total bronchoalveolar lavage surfactant phosphatidylcholine (A) and fractional** ***methyl*** **-D**_**9**_ **PC16:0/16:0 (B), PC16:0/14:0 (C), PC16:0/16:1 (D), PC16:0/18:2 (E) and PC16:0/18:1 (F) enrichment in patients and controls.** Data presented as mean ± SEM, †, P < 0.05. PC, phosphatidylcholine.

Despite the lower concentration of BALF PC in ARDS patients compared to controls (Figure 2), the fractional enrichments of *methyl*-D_9_-choline into both total PC and PC16:0/16:0 were consistently higher in patients compared with healthy controls (Figure 5). Expressed as a percentage of total labelled plus endogenous PC, the fractional enrichment of *methyl*-D_9_-choline into total PC for patients increased from 0.19 ± 0.05% at 6 hours to a maximum value of 0.90 ± 0.2% at 48 hours and then remained constant until 96 hours. Compared to controls, this represented 77% and 73% increases in total PC enrichment at 24 and 48 hours respectively (Figure 5A).

The fractional PC16:0/16:0 *methyl*-D_9_ enrichment for patients reflected that of total PC, but with a time delay. PC16:0/16:0 *methyl*-D_9_ enrichment at 6 hours (0.09 ± 0.03%) was much lower than total PC enrichment for this time point and maximal enrichment was not observed until 72 hours (0.79 ± 0.08%) (Figure 5B). Although, compared to healthy controls *methyl*-D_9_-PC16:0/16:0 enrichments were higher in patients, statistical significance (P < 0.05) was only noted at 48 hours after *methyl*-D_9_ choline infusion. Other major PC species also showed significant increases in the fractional *methyl*-D_9_ enrichment compared to healthy controls (Figure 5C, D, E, and F).

**Figure 6:**
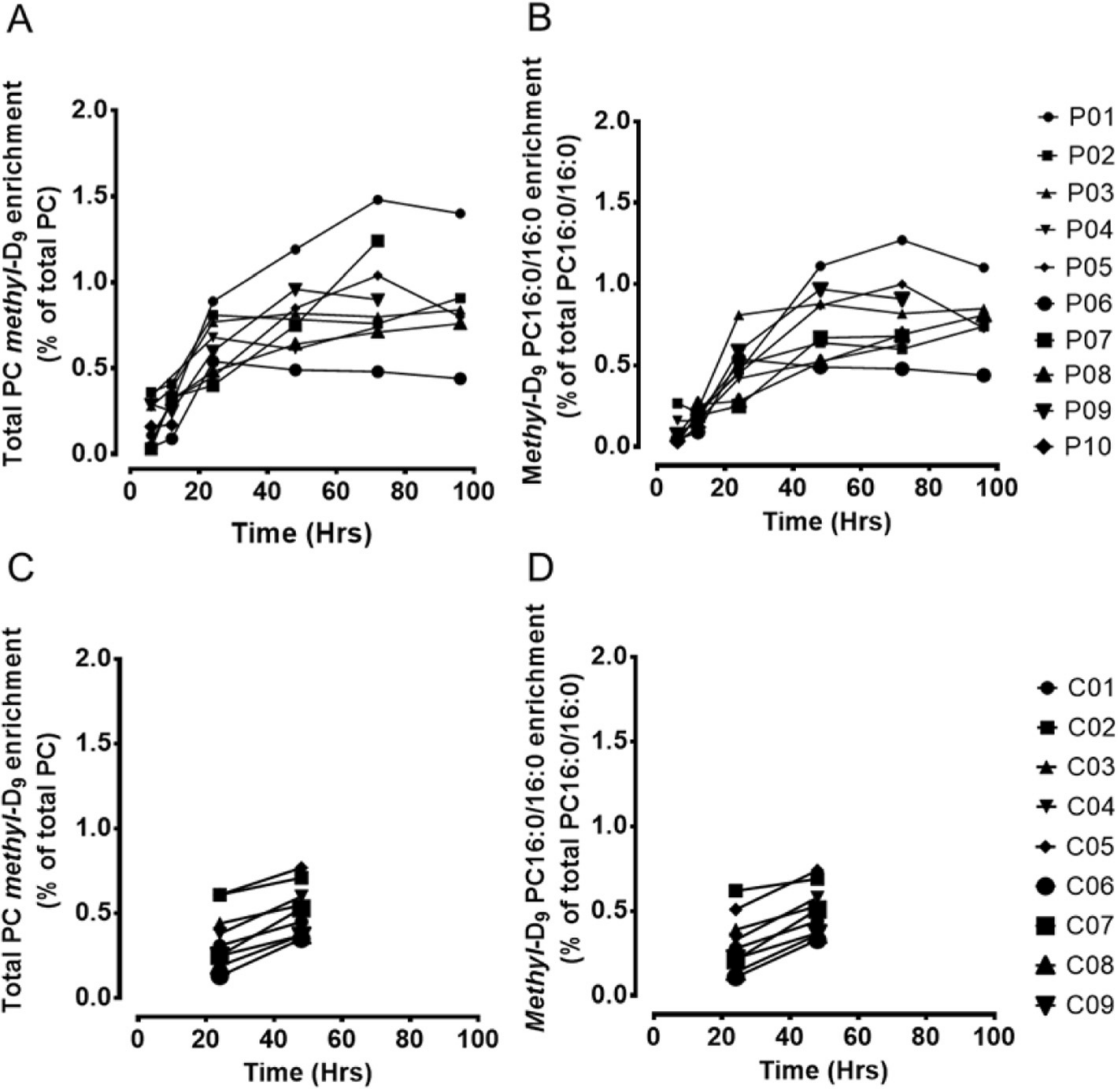
**Bronchoalveolar lavage surfactant total phosphatidylcholine and fractional PC16:0/16:0 enrichment patterns for individual patients and controls. A**, Individual variation in the total PC *methyl*-D_9_ enrichment among patients; **B**, Individual variation in the PC16:0/16:0 *methyl*-D_9_ enrichment among patients; **C**, Individual variation in the total PC *methyl*-D_9_ enrichment among controls; **D**, Individual variation in the PC16:0/16:0 *methyl*-D_9_ enrichment among controls.

**Figure 7:**
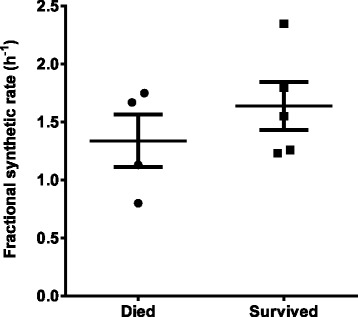
**Fractional synthetic rate (FSR) of phosphatidylcholine enrichment in BALF samples from patients with ARDS.** Results were calculated as the initial gradient of enrichment divided by the plateau enrichment expressed as a percentage. While there was a three-fold range in values, there was no significant difference for those who survived (mean = 1.64 h^−1^) and those patients who died (mean = 1.40 h^−1^, P = 0.36).

However, these mean enrichment values masked wide variations for individual patients in both magnitude of label enrichment and the time required to achieve maximal enrichment for both total *methyl*-D_9_-PC (Figure 6A) and *methyl*-D_9_-PC16:0/16:0 (Figure 6B). For instance, the time of maximal enrichments for both parameters varied from 24 to 72 hours for individual patients. While the corresponding enrichments for controls at both 24 and 48 hours exhibited a similar variation to patients (Figure 6C and 6D), we were unable to determine their variation in time to maximal enrichment. Unfortunately, without measurement of the isotopic enrichment of the *methyl*-D_9_ label in the phosphocholine substrate pool within AT- II cells, no calculation of a fractional synthetic rate (FSR) from the initial slope of enrichment can account for individual variation in substrate isotopic enrichment. An alternative approach to calculate FSR from the initial gradient of isotopic enrichment divided by the plateau value displayed a three-fold variation for the 9 patients who survived beyond 24 h, but values for those patients who died was not significantly different from those who survived (Figure 7). Over the 96 hours duration of this study, there was no substantial decrease in *methyl*-D_9_ choline enrichment into BALF PC in the patient group, precluding any calculation of fractional catabolic rate (FCR).

### Molecular composition of newly synthesised PC species

**Figure 8:**
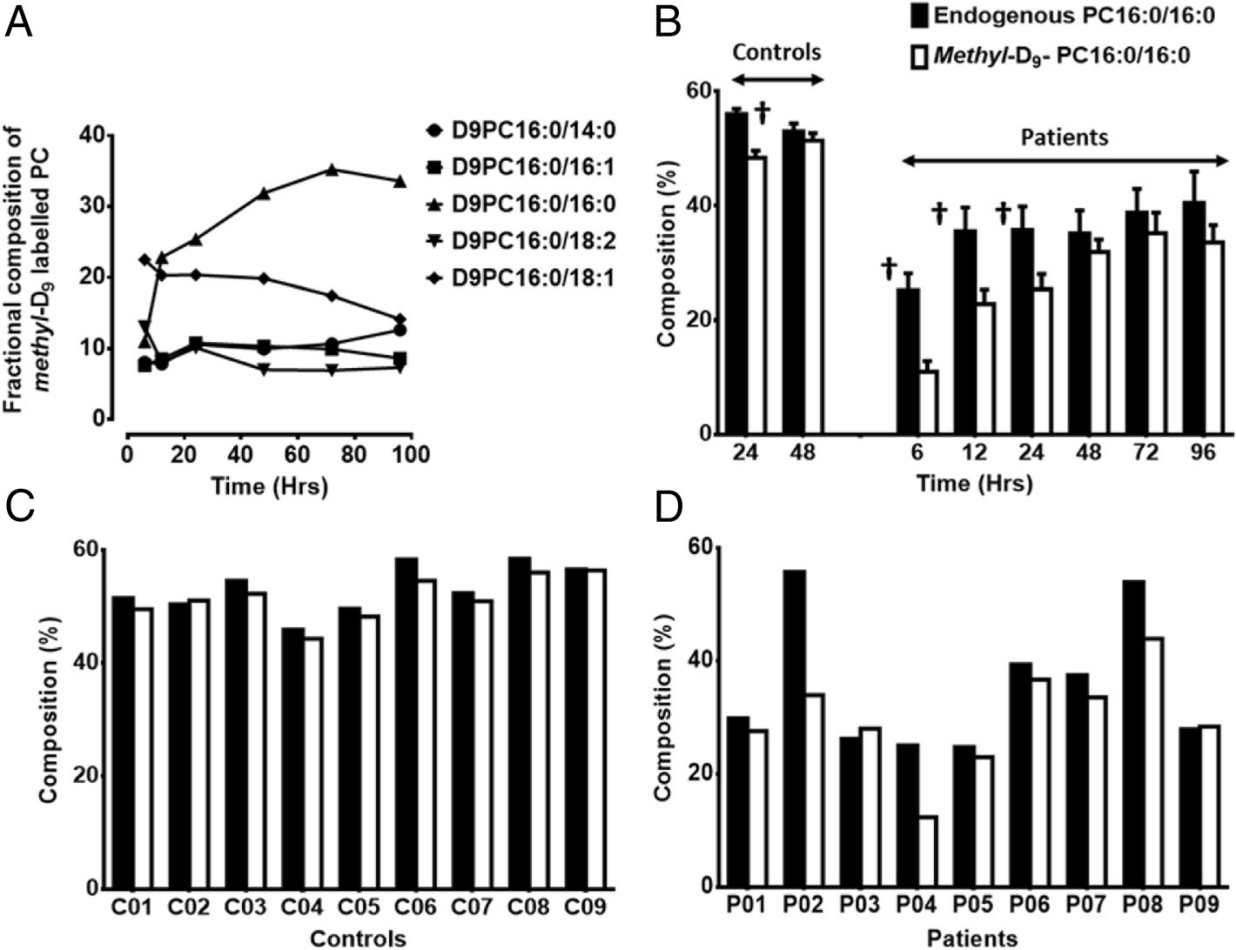
**Molecular specificity of newly synthesised major bronchoalveolar lavage surfactant phosphatidylcholine fraction in ARDS patients over time (A), endogenous and** ***methyl*** **-D**_**9**_ **labelled fractional composition of PC16:0/16:0 for both controls and patients (B), endogenous and** ***methyl*** **-D**_**9**_ **labelled fractional composition of PC16:0/16:0 for individual controls (C) and patients (D) at 48 hours after** ***methyl*** **-D**_**9**_ **-choline infusion.** At the earliest time point (T = 06 Hrs) after *methyl*-D_9_ choline infusion, unsaturated PC species predominate with subsequent gradual increase in PC16:0/16:0 over time. PC16:0/16:0 synthesis, expressed as a percentage of newly-synthesised total PC, was compared with that of endogenous PC16:0/16:0 at two time points for controls and six time points for patients. The proportion of *methyl-*D_9_-labelled PC16:0/16:0 was significantly lower than that of unlabelled PC16:0/16:0, when both are expressed relative to total labelled and unlabelled PC pools, and at 24 hours this deficit is greater in ARDS compared with control subjects. For both patients and controls, the newly synthesised fraction of PC16:0/16:0 was in equilibrium with endogenous composition at 48 hours after *methyl*-D_9_ choline infusion. Presented as mean ± SEM; † P < 0.05. There is considerable variation in the relative proportion of fractional labelling of PC16:0/16:0 in patients compared to the controls in addition to lower fractional synthesis of PC16:0/16:0 in 3 patients (P02, P04 and P08) compared with endogenous fraction. Controls (n) =9 and patients (n) =9. PC, phosphatidylcholine. C, individual controls; P, individual patients.

The molecular composition of newly synthesised and secreted PC species in BALF from patients, quantified from precursor scans of P193 (Figure 1), varied considerably with time after *methyl-*D_9_-choline infusion (Figure 8A). PC synthesis composition at the earliest time point (T = 06 Hrs) after *methyl-*D_9_ choline infusion was dominated by the unsaturated species PC16:0/18:1 (22.5 ± 3.6%) and PC16:0/18:2 (13.1 ± 1.8%). The fractional labelling of PC16:0/16:0 gradually increased from 11 ± 1.9% at 6 hours to 25.4 ± 2.7%, 32 ± 2.2%, 35.2 ± 3.6% and 33.6 ± 3.9% of total labelled PC at 24, 48, 72 and 96 hours respectively, accompanied by a corresponding decreased fractional labelling of unsaturated PC species, particularly PC16:0/18:1 from 22.5 ± 3.6% at 6 h to 14.2 ± 4.1% at 96 hours after choline infusion (Figure 8A).

In patients, the gradual increase of fractional labelled PC16:0/16:0 lagged behind the improvement of endogenous PC16:0/16:0 composition with time. At the earliest time point (T = 06 Hrs) after *methyl-*D_9_ choline infusion, the newly synthesised fraction of PC16:0/16:0 was significantly lower than that of the endogenous fraction (newly synthesised 11 ± 1.9% versus endogenous 25.2 ± 2.9%), but achieved near equilibrium by 48 hours (Figure 8B). For healthy controls, *methyl*-D_9_ enrichment into BALF PC was only determined at 24 and 48 hours. Comparing patients and controls at 24 hours, while fractional labelling of PC16:0/16:0 in healthy controls was lower than endogenous composition (48.4 ± 1.2% vs 56.1 ± 0.9%), this difference was significantly greater for the patient group (25.4 ± 2.7 vs 35.8 ± 4.1%). However, similar to patients, the *methyl*-D_9_ labelled PC16:0/16:0 achieved near equilibrium with endogenous composition of PC16:0/16:0 at 48 hours in the control group (Figure 8B). In contrast to the small variation in the percentage composition of newly synthesised PC16:0/16:0 for the healthy controls, this value varied considerably for the patients, ranging from 15-47% of total *methyl*-D_9_-labelled PC (Figure 8C and 8D). However, with the exception of three patients (P02, P04 and P08), who had continued lower fractional composition of *methyl*-D_9_-labelled PC16:0/16:0, this variation in patients reflected the variation of endogenous fractional composition of PC16:0/16:0 at 48 hours (at overall peak PC *methyl*-D_9_-enrichment).

## Discussion

This study conducted on a small number of ARDS patients illustrates the feasibility of performing stable isotope labelling in combination with ESI-MS/MS analytical methods to investigate surfactant phospholipid metabolism in a defined human patient cohort. This is the first study to characterise the molecular specificity of surfactant synthesis in adult ARDS population. The study group composed of patients with moderate to severe ARDS as defined by the degree of hypoxemia. Most patients (90%) had pneumonia as the single precipitating factor.

Despite the significant variability, ARDS patients had persistently lower BALF total PC concentrations compared to healthy controls. The BALF fractional PC16:0/16:0 absolute concentrations were also much lower in patients and only accounted for about 10% of the healthy controls. The decreased fractional PC16:0/16:0 content in our patient group is comparable with a previous study of patients with ARDS, where the disaturated PC content of from patients was only 17% of that of controls [11]. Apparent differences with other previous reports in ARDS are largely due to a combination of less severe disease [5] and alternative methodologies and analysis of purifying isolated surfactant fractions [2,5,12]. This deficit in total PC and fractional PC16:0/16:0 concentration may be due to reduced synthesis, increased breakdown, or dilution by pulmonary oedema. Reduced synthesis in turn could be due to either destruction of AT-II cells or dysfunction of their synthesis and secretory mechanisms. However, this was coupled with increased *methyl*-D_9_ choline enrichments of both total PC and PC16:0/16:0 (Figure 5A and 5B). One possible explanation for this finding is that the rate of surfactant PC synthesis in remaining uninjured AT-II cells must be preserved or even enhanced, but that total surfactant PC production is indeed decreased probably due to decreased numbers of functional AT-II cells. This conclusion support and extend a previous study that employed in-vivo stable isotope labelling with deuterated water (^2^H_2_0) to characterise patterns of enrichment of newly-synthesised fatty acids into disaturated PC in ARDS patients [11]. This study also demonstrated increased fractional labelling of disaturated PC, coupled with decreased concentration, but provided no specific molecular species analysis. In this context, the interaction between rates of PC synthesis on the endoplasmic reticulum, its packaging into lamellar bodies and subsequent secretion will be critical. One realistic scenario, for instance, could be that decreased surfactant secretion from a depleted number of AT-II cells results in accumulation of labelled PC in existing AT-II cells, which could explain the conundrum of decreased concentration, but increased fractional enrichment of surfactant PC in ARDS patients.

As fractional catabolic rates could not be calculated for either ARDS patients or healthy controls, no conclusion can be inferred about the potential contribution of increased surfactant catabolism to decreased BALF PC concentration in ARDS patients. However, any substantial increased surfactant catabolism would have been expected to cause a more rapid decline in PC enrichment in ARDS patients compared with control subjects. While such a comparison was not possible in the present study, the duration of labelling of sputum PC in previous studies [8,13] was very similar to the enrichment profiles of BALF PC in ARDS patients shown in Figure 5. Consequently, the most likely cause of the lower BALF PC concentration in ARDS patients was a significantly decreased rate of total alveolar surfactant synthesis.

The newly synthesised surfactant fraction in ARDS patients also revealed abnormal PC composition, with diminished levels of PC16:0/16:0 and increased fractional compositions of unsaturated PC species, when compared to the endogenous composition. We have previously demonstrated in healthy volunteers that, during earlier time points following *methyl*-D_9_-choline infusion, the newly synthesised surfactant PC complex is different to that of endogenous composition [8,13]. Furthermore, it takes up to 48 hours for the labelled *methyl*-D_9_-PC16:0/16:0 to achieve equilibrium with unlabelled PC16:0/16:0 percentage composition. If the acyl- remodelling mechanisms described previously [8,9,13] are complete before surfactant secretion, the labelled PC16:0/16:0 composition should be at equilibrium with endogenous composition at all-time points. As our data shows this not be the case, our results are compatible with the previous healthy volunteer study conclusion, that not all alveolar surfactant is acyl- remodelled prior to secretion [8,13]. Despite similarities, ARDS patients had much lower fractional composition of labelled PC16:0/16:0 at all- time points compared to healthy controls. In-addition, three patients had comparatively much lower fractional labelling compared to the endogenous fraction of PC16:0/16:0 at 48 hours. These findings indicate, that despite an increase in fractional synthesis, the surfactant PC produced has abnormal composition with some patients showing altered acyl-remodelling mechanisms of PC16:0/16:0.

Previous surfactant related clinical studies used gas chromatographic separation methods to quantify palmitic acid (16:0) and used the latter as a surrogate for PC16:0/16:0 [2,5]. This methodology always produced higher fractional compositional values (70-80%) for palmitic acid (16:0), which may have come from PC species other than PC16:0/16:0, and have no clinical relevance. In contrast, stable isotope labelling of surfactant precursors in combination of ESI-MS/MS analytical approach, is a novel technique to study dynamic phospholipid flux with precise molecular analysis. This facilitated for the first time the assessment of surfactant PC molecular synthesis via CDP-choline pathway in adult ARDS population.

This is the first study to use small volume BALF to investigate surfactant metabolism in patients with ARDS. In the past, investigators have used quantitative BALF to investigate compositional analysis in ARDS. Although in general this is a safe procedure, repetitive large volume BALF may theoretically deplete alveolar surfactant and may produce a negative clinical impact. Moreover, anecdotally desaturations are much more common during quantitative BALF. The surfactant PC composition in our control group is similar to previous publications, which has used large volume BALF for molecular analysis [12,14]. This suggests, at least in physiological conditions small volume BALF is comparable to quantitative BALF for the study of surfactant phospholipid composition and metabolism. Small volume BALF was tolerated by all, without any significant immediate complications. Despite no cardiovascular or respiratory compromise, all patients needed additional sedation, on average of 40 mgs of propofol or 2 mgs of midazolam to perform this procedure.

As this was a pilot study with small number of patients, no clinical correlations or outcome inferences were made. The study was further limited by the lack of ventilated control patients, who are more representative as a comparable population, than healthy adults. Despite these limitations, the demonstration of the existence of variable surfactant composition, synthesis and metabolism among patients indicate, the presence of underlying phenotypes of surfactant metabolism. Future similar studies if adequately powered, may help to explore clinical correlations.

## Conclusions

This preliminary data suggest the feasibility of using isotope labelling technique to study surfactant kinetics in patients with ARDS. The compositional assessments indicate variability in the surfactant PC composition, fractional synthesis and enrichment of newly synthesised surfactant PC species among patients. This individual variability may pave the way for phenotypic characterisation of patients according to surfactant metabolism and subsequent therapeutic targets.

## Abbreviations

APACHE II score: Acute physiology and chronic health evaluation
ARDS: Acute respiratory distress syndrome
AT-II cells: Alveolar type II cells
BALF: Bronchoalveolar lavage fluid
BHT: Butylated hydroxyl toluene
CDP: Cytidine diphosphate
ESI-MS/MS: Electrospray ionisation mass spectrometry
Hrs: Hours
IBW: Ideal body weight
ICU: Intensive care unit
PC: Phosphatidylcholine
SatPC: Saturated phosphatidylcholine
